# 589. HEAL-CEA One Burn Center's Multidisciplinary Approach to CEA Care

**DOI:** 10.1093/jbcr/irag033.383

**Published:** 2026-04-06

**Authors:** Shannon Bing, Patrick Brockway, Mahmoud Hassouba, David M Hill, Sarah E Sabbatini

**Affiliations:** Firefighters Regional One Burn Center, Memphis, TN; Firefighters Regional One Burn Center, Memphis, TN; Firefighters Regional One Burn Center, Memphis, TN; Firefighters Regional One Burn Center, Memphis, TN; Firefighters Regional One Burn Center, Memphis, TN

## Abstract

**Introduction:**

Cultured epidermal autografts (CEA) offer a critical reconstructive option for patients with extensive burn injuries and limited donor sites. However, standardized postoperative rehabilitation protocols remain lacking, particularly regarding adjunctive therapies such as phototherapy. This review evaluates the role of interdisciplinary collaboration in optimizing outcomes following CEA application.

**Methods:**

A retrospective chart review was conducted for seven patients treated with CEA at a regional burn center between 2021 and 2023. Data included injury characteristics, team coordination practices, timing of phototherapy, complications, and functional outcomes.

**Results:**

Patients had TBSA burns ranging from 38% to 70%, with an average hospital stay of 84.2 days. Graft adherence ranged from 90% to 100% (mean 95.4%).phototherapy was universally applied, typically initiated between post-operative day (POD) 1 and 4. In 80% of cases, patients returned to pre-burn activity levels. Interdisciplinary coordination was linked to consistent therapy application, early complication identification, and improved patient education.

**Conclusions:**

Multidisciplinary collaboration improved postoperative outcomes in CEA patients, even in the absence of standardized protocols. Structured team communication and coordinated phototherapy contributed to high graft adherence and functional recovery. These findings support the development of evidence-based, team-driven care models.

**Applicability of Research to Practice:**

Interdisciplinary teamwork and early phototherapy are key to successful CEA recovery. Consistent team communication, coordinated therapy schedules, and patient/family education improve graft adherence and functional outcomes. These findings support the development of structured, team-driven protocols to guide postoperative burn rehabilitation.

**Funding for the study:**

N/A.

